# Increasing Systemic Immune-inflammation Index During Treatment in Patients With Advanced Pancreatic Cancer is Associated With Poor Survival: A Retrospective, Multicenter, Cohort Study

**DOI:** 10.1097/SLA.0000000000005865

**Published:** 2023-04-03

**Authors:** Freek R. van ‘t Land, Mohammad H. Aziz, Nynke Michiels, J. Sven D. Mieog, Bert A. Bonsing, Saskia A.C. Luelmo, Marjolein Y.V. Homs, Bas Groot Koerkamp, Grigorios Papageorgiou, Casper H.J. van Eijck

**Affiliations:** * Department of Surgery, Erasmus MC Cancer Institute, Rotterdam, the Netherlands; † Department of Surgery, Leiden University Medical Center, Leiden, the Netherlands; ‡ Department of Oncology, Leiden University Medical Center, the Netherlands; § Department of Medical Oncology, Erasmus MC Cancer Institute, Rotterdam, the Netherlands; ∥ Department of Biostatistics, Erasmus MC University Medical Center, Rotterdam, the Netherlands; ¶ Department of Epidemiology, Erasmus MC University Medical Center, Rotterdam, the Netherlands

**Keywords:** FOLFIRINOX chemotherapy, inflammation, pancreatic cancer, stereotactic body radiotherapy, systemic immune-inflammation index, survival

## Abstract

**Background and Objectives:**

A high systemic immune-inflammation index (SIII) at diagnosis of various cancers, including pancreatic cancer, is associated with poor prognosis. The impact of FOLFIRINOX (5-fluorouracil, leucovorin, irinotecan, and oxaliplatin) chemotherapy or stereotactic body radiotherapy on this index is unknown. In addition, the prognostic value of changes in the SIII during treatment is unclear. In this retrospective analysis, we aimed to find answers regarding patients with advanced pancreatic cancer.

**Methods:**

Patients with advanced pancreatic cancer treated with FOLFIRINOX chemotherapy alone or with FOLFIRINOX chemotherapy followed by stereotactic body radiotherapy between 2015 and 2021 in 2 tertiary referral centers were included. Baseline characteristics, laboratory values at 3 time points during treatment, and survival outcomes were collected. The patient-specific evolutions of SIII and their association with mortality were assessed with joint models for longitudinal and time-to-event data.

**Results:**

Data of 141 patients were analyzed. At a median follow-up time of 23.0 months (95% CI: 14.6–31.3), 97 (69%) patients had died. Median overall survival was 13.2 months (95% CI: 11.0–15.5). During treatment with FOLFIRINOX, the log (SIII) was reduced by −0.588 (95% CI: −0.0978, −0.197; *P* = 0.003). One unit increase in log (SIII) increased the hazard ratio of dying by 1.604 (95% CI: 1.068–2.409; *P* = 0.023).

**Conclusions:**

In addition to carbohydrate antigen 19-9, the SIII is a reliable biomarker in patients with advanced pancreatic cancer.

Pancreatic ductal adenocarcinoma (PDAC) is a highly lethal malignancy. By 2030, it is anticipated to rank as the second leading cause of cancer-related fatalities in the United States.^1^ When diagnosed, the cancer is anatomically staged as resectable, borderline resectable, locally advanced, or metastasized pancreatic cancer. Unfortunately, due to the lack of symptoms and rapid progression only 10% to 20% of patients present with resectable or borderline resectable tumors, whereas the vast majority present with locally advanced pancreatic cancer (LAPC) or metastasized pancreatic cancer.^2^ As available treatments have only slightly improved survival over the past decades,^3^ it is crucial to find better prognostic biomarkers during various types of treatment. This will aid clinicians in making better treatment decisions, and prevent futile treatment.

Systemic chemotherapy is the first-line treatment option for LAPC or metastasized PDAC, preferably with FOLFIRINOX (5-fluorouracil, leucovorin, irinotecan, and oxaliplatin).^2^ In 2011, a randomized clinical trial clearly demonstrated longer survival in patients with metastasized PDAC treated with the multiagent regimen FOLFIRINOX compared with single-agent gemcitabine.^4^ Unfortunately, this improved efficacy was accompanied by toxicity, especially neutropenia, diarrhea, and peripheral neuropathy.^4,5^ After successful treatment with FOLFIRINOX in LAPC patients, stereotactic body radiotherapy (SBRT) can be added to the treatment regimen.^6–9^ Multiple single-arm studies have been published treating patients with SBRT, but data from randomized clinical trials are lacking. With the introduction of FOLFIRINOX chemotherapy, outcomes for patients with pancreatic cancer have improved, but the prognosis is still dismal. For this reason, and as well as considering the toxicity of the available chemotherapy, the need to find prognostic biomarkers is evident.

Currently, carbohydrate antigen (CA) 19-9 is the most-used serological biomarker.^10,11^ However, 10% to 20% of patients with pancreatic cancer do not have elevated levels of CA 19-9.^10,12^ In recent years, the role of cancer-associated inflammation in carcinogenesis, cancer progression, and prognosis has increasingly been recognized.^13^ The systemic immune-inflammation index (SIII) is an easily obtainable biomarker reflecting the extent of systemic inflammation. The SIII has been shown to be an independent predictor of cancer-specific survival and recurrence in patients with resectable pancreatic cancer.^14^ Also, in advanced pancreatic cancer patients treated with first-line chemotherapy, elevated SIII at the time of diagnosis was found an independent risk factor for impaired overall and progression-free survival.^15^ Thus, SIII is a promising biomarker in PDAC. It is not known, however, whether and how the SIII is influenced during treatment with novel therapies such as FOLFIRINOX or SBRT, and what the possible prognostic value of changes in the SIII is. Therefore, the primary objective of this study was to evaluate whether longitudinal monitoring of the SIII during treatment with FOLFIRINOX alone or with FOLFIRINOX followed by SBRT has prognostic value in patients with advanced PDAC.

## METHODS

### Patients

We retrospectively assessed data of all patients with advanced pancreatic cancer who were treated with FOLFIRINOX with or without consecutive SBRT between 2015 and 2021 in 2 tertiary referral centers (Erasmus University Medical Center and Leiden University Medical Center). Inclusion criteria were (1) LAPC patients according to Dutch Pancreatic Cancer Group criteria for resectability (ie, >90 degrees tumor contact with the superior mesenteric artery, the celiac trunk, and/or any hepatic artery, and/or >270 degrees tumor contact with the superior mesenteric vein and/or the portal vein or venous occlusion)^16^ or patients with proven metastatic disease and (2) treatment with FOLFIRINOX with or without consecutive SBRT. Exclusion criteria were (1) resectable or borderline resectable pancreatic cancer at diagnosis and (2) treatment with chemotherapy other than FOLFIRINOX. Patients with nonprogressive LAPC and with insufficient drop in CA 19-9 (<50%) after FOLFIRINOX, were eligible to receive consecutive SBRT. The study was approved by the medical ethical committees of the participating hospitals (Erasmus University Medical Center study number: MEC-2022-0138; Leiden University Medical Center study number: G17.059). Because of the retrospective nature of this study and the poor prognosis, a waiver of informed consent was approved by the ethical committees. The reporting adhered to the “Strengthening the Reporting of Observational Studies in Epidemiology” guidelines.^17^

### Data Collection

We collected all relevant variables from the local electronic patient files. Patient characteristics [ie, sex, age at the start of chemotherapy, length (cm), weight (kg), and disease stage (LAPC or metastasized)] were collected. Relevant laboratory assessments, CA 19-9 (U/mL), carcinoembryonic antigen (µg/L), absolute platelet count (×10^9^/L), absolute neutrophil count (×10^9^/L), absolute lymphocyte count (×10^9^/L), total bilirubin levels (µmol/L, mg/dl), and total leukocyte counts (×10^9^/L) were collected. The SIII was calculated by multiplying the neutrophil count with the platelet count and dividing the result by the lymphocyte count (N×*P*/L). Laboratory values were collected at 3 time points. Time point 1 = before the start of FOLFIRINOX treatment, time point 2 = after FOLFIRINOX treatment, and before the start of SBRT, and time point 3 = after SBRT. Time point 3 values were not available for patients who had not been treated with SBRT after FOLFIRINOX. Oncological treatment characteristics collected were the start date and stop date of chemotherapy, and the number of cycles of chemotherapy. Radiotherapy treatment characteristics collected were the type of radiotherapy, start date and stop date of radiotherapy, and amount of Gray. Surgical treatment characteristics were the number of resections, date of resection, and histopathological outcomes. In addition, the date of death or the date last known to be alive was collected.

### Main Endpoint

The main study endpoint was the overall survival (OS), defined as the time interval between the day of starting FOLFIRINOX chemotherapy and the date of death or date last known to be alive.

### Statistical Analyses

Baseline patient characteristics are summarized using the median and interquartile range (IQR) for continuous variables and using counts and percentages for categorical variables. The median follow-up time was calculated using the reverse Kaplan-Meier method. The median OS was estimated using the Kaplan-Meier method. Patients alive were censored at the last follow-up. Survival curves were statistically compared using log-rank tests. To assess the effect of changes in the SIII on the instantaneous risk of dying during treatment with FOLFIRINOX alone or treatment with FOLFIRINOX and SBRT, a joint model for longitudinal and time-to-event data was used. First, to estimate the patient-specific trajectories of SIII, the repeated measurements of SIII were analyzed using a mixed-effects model with random intercepts and slopes. A log transformation of SIII was used to fulfill the model assumptions of normality and homoscedasticity. The model included time-varying effects of FOLFIRINOX and SBRT to capture changes in the trajectories of SIII due to treatment. The estimated patient-specific trajectories then served as time-varying covariates in a relative risk model, which was adjusted for baseline body mass index, baseline age, and sex to capture the association between the evolution of SIII and the risk of dying.^18–21^ Similar analyses were performed for CA 19-9, neutrophils, lymphocytes, and platelets. All statistical analyses were performed using SPSS Statistics version 25 and *R* version 4.1.2.

## RESULTS

### Patient Characteristics

Data of 141 patients were included. Sixty-three (45%) were males and the median age was 63 (IQR 56–69) years. The median body mass index was 24 (IQR: 22–28) kg/m^2^. At baseline, median CA 19-9 was 248 (IQR: 60–1376) U/mL, median carcinoembryonic antigen was 4.2 (IQR: 2.9–11.7) µg/L, median SIII was 908 (IQR: 632–1276), and the median total bilirubin value was 13.5 (IQR: 7.0–25.8) µmol/L (0.15 (IQR: 0.08– 0.29) mg/dL). Fifty (35.4%) patients were treated with FOLFIRINOX alone, and 91 (64.5%) patients were treated with FOLFIRINOX and consecutive SBRT. Detailed patient characteristics are shown in Table 1.

**TABLE 1:** Patient Characteristics

Baseline Characteristics	N = 141; n (%)
Age (yr), median (IQR)	63 (56–69)
Sex (M)	63 (45)
BMI (kg/m^2^), median (IQR)	24 (22–28)
CA 19-9 (U/mL), median (IQR)	248 (60–1376)
CEA (µg/L), median (IQR)	4.2 (2.9–11.7)
Bilirubin (µmol/L), median (IQR)	13.5 (7.0–25.8)
CRP (mg/L), median (IQR)	4.1 (1.9–11.3)
G-CSF	
Yes	82 (58)
Unknown	59 (42)
Disease stage	
LAPC	118 (84)
Metastasized pancreatic cancer	23 (16)
Oncological treatment characteristics	
FOLFIRINOX alone	50 (35)
FOLFIRINOX and consecutive SBRT	91 (65)
No. of cycles, median (IQR)	8 (6–8)

Continuous variables are shown as medians with the IQR.

Categorical variables are shown as counts with percentages.

BMI indicates body mass index; CEA, carcinoembryonic antigen; CRP, *C*-reactive protein.

### Laboratory Values

Laboratory values were collected at 3 different time points. The SIII value was available at time point 1 (ie, before the start of FOLFIRINOX) in 89/141 (63.1%) patients, at time point 2 (ie, after FOLFIRINOX and before SBRT) in 57/141 (40.4%) patients, and at time point 3 (ie, after SBRT) in 56/91 (61.5%) patients. In 117/141 (83.0%) patients the SIII value was available in at least one of the 3 time points. The median time between time point 1 and the start of FOLFIRINOX chemotherapy was 8 (IQR: 0–33) days. The median time between the stopping of FOLFIRINOX and time point 2 was 21 (IQR: 8–47) days. The median time between the end of SBRT and time point 3 was 10 (IQR: 10 –100) days. Detailed laboratory data availability is shown in Supplementary Table 1 (Supplemental Digital Content 1, http://links.lww.com/SLA/E512).

### Changes in the Systemic Immune-inflammation Index After Folfirinox and Stereotactic Body Radiotherapy

**FIGURE 1:**
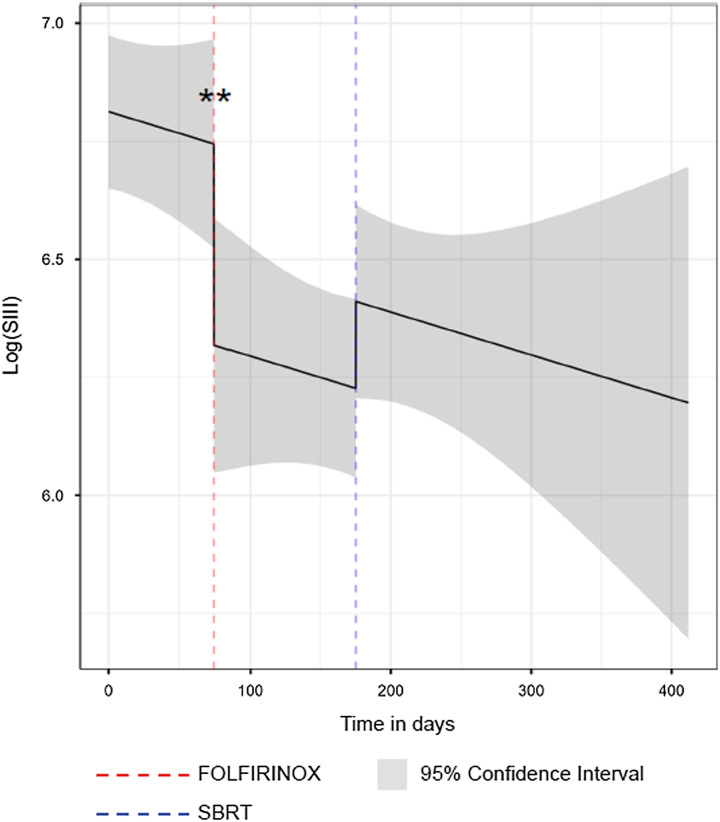
Average longitudinal trajectory of the log (SIII) during treatment. Joint model analysis of the longitudinal trajectory of the SIII demonstrates that the SIII was significantly reduced after treatment with FOLFIRINOX chemotherapy. The SIII before and after SBRT was not different. Statistical significance of “**” indicates a *P* value <0.01.

The median SIII at the first time point (before the start of FOLFIRINOX) was 908 (IQR: 632– 1276). The median SIII at the second time point (after FOLFIRINOX) was 488 (IQR: 352–691), and the median SIII at the third time point (after SBRT) was 536 (IQR: 398–832) (Supplemental Digital Content Table 2, http://links.lww.com/SLA/E513). There was no difference in the baseline SIII between LAPC and metastasized PDAC patients [LAPC vs metastasized PDAC; 905 (IQR: 645–1318) vs 1039 (IQR: 540–1249); *P* = 0.930]. The joint model analysis showed that after treatment with FOLFIRINOX, the log (SIII) had decreased by −0.588 (95% CI: −0.978, −0.197; *P* = 0.003). The log (SIII) had increased by 0.167 after treatment with SBRT, but without statistical significance (95% CI: −0.059, 0.393; *P* = 0.148). Figure 1 demonstrates the average longitudinal trajectory of the log (SIII). After FOLFIRINOX, lymphocytes had not changed, but we found an increase in neutrophils and a decrease in platelets. After SBRT, platelets had not changed, but we observed a decrease in lymphocytes and neutrophils. These data are presented in detail in Table 2.

**TABLE 2:** Effect of FOLFIRINOX and SBRT on the SIII, Neutrophils, Lymphocytes, Platelets, and CA 19-9

		FOLFIRINOX			SBRT		
Variable	n	Change	95% CI	*P*	Change	95% CI	*P*
Log (SIII)	117	−0.588	−0.978, −0.197	**0.003**	+0.167	−0.059, 0.393	0.148
Log (neutrophils) (×10^9^/L)	132	+0.229	0.009, 0.465	**0.041**	−0.311	−0.515, −0.107	**0.003**
Log (lymphocytes) (×10^9^/L)	117	−0.14	−0.392, 0.112	0.276	−0399	−0.569, −0.229	**<0.001**
Platelets (×10^9^/L)	136	−114	−143, −84	**<0.001**	+9.686	−16.619, 35.991	0.470
CA 19-9 (U/mL)	132	+0.468	−0.087, 1.023	0.098	+0.343	−0.004, 1.023	0.053
CA 19-9^*^ (U/mL)	105	−0.562	−1.185, 0.06	0.077	+0.028	−0.424, 0.48	0.903

Bold *P* value indicates statistical significance (P < 0.050).

* Analysis of CA 19-9 in patients with elevated CA 19-9.

n indicates number of patients included in the analysis. If a variable is available in at least one of the 3 time points, this patient is included in the analysis. In some variables, data are log-transformed to not violate the 2 joint model assumptions (normality of residuals and homoscedasticity).

### Systemic Immune-inflammation Index as a Prognostic Biomarker for Overall Survival During Treatment

**FIGURE 2:**
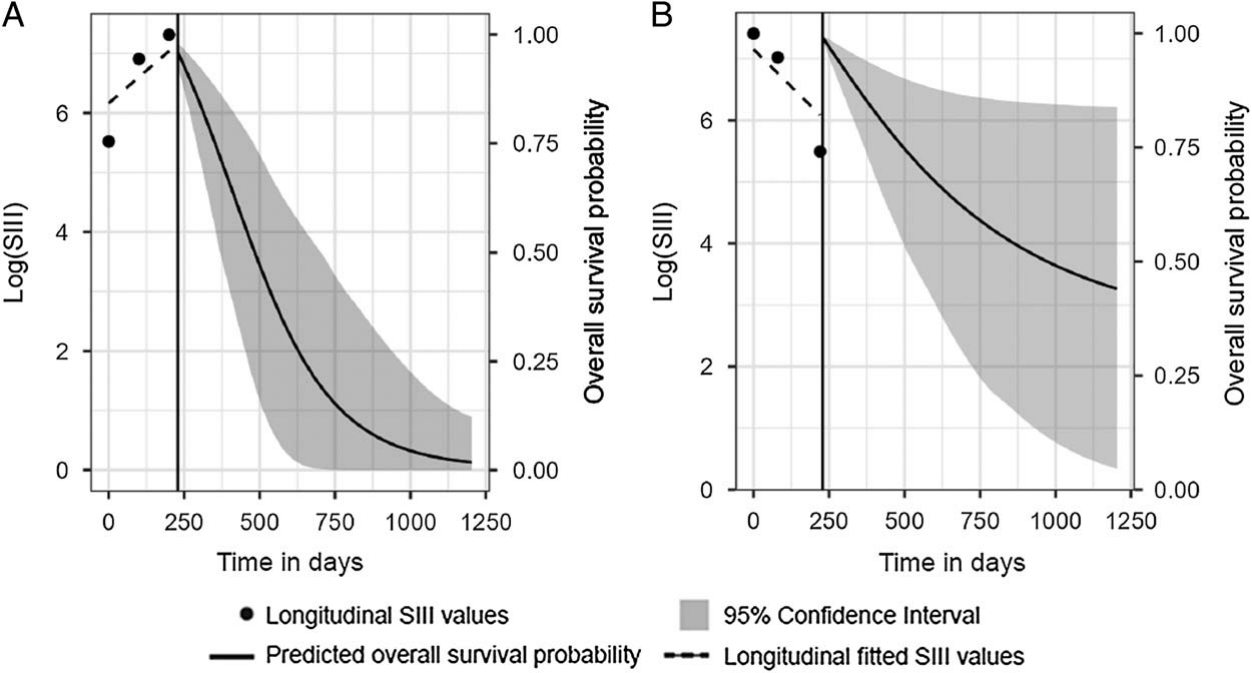
Increasing SIII values were associated with worse OS. A, An example of a patient with increasing SIII values during treatment and the associated OS probability. B, An example of a patient with decreasing SIII values during treatment and the associated OS probability. Increasing SIII values during treatment were associated with worse survival.

The median follow-up length from the start of FOLFIRINOX to the last follow-up was 23.0 months (95% CI: 14.6– 31.3). Ninety-seven (69%) patients had died during follow-up. The median OS was 13.2 months (95% CI: 11.0–15.5). Joint model analysis for the association between the SIII and the instantaneous risk of death showed that one unit increase in log (SIII) increased the hazard ratio of dying by 1.604 (95% CI: 1.068, 2.409; *P* = 0.023). Figure 2 demonstrates the difference in OS probability in 2 patients with different longitudinal SIII trajectories.

**FIGURE 3:**
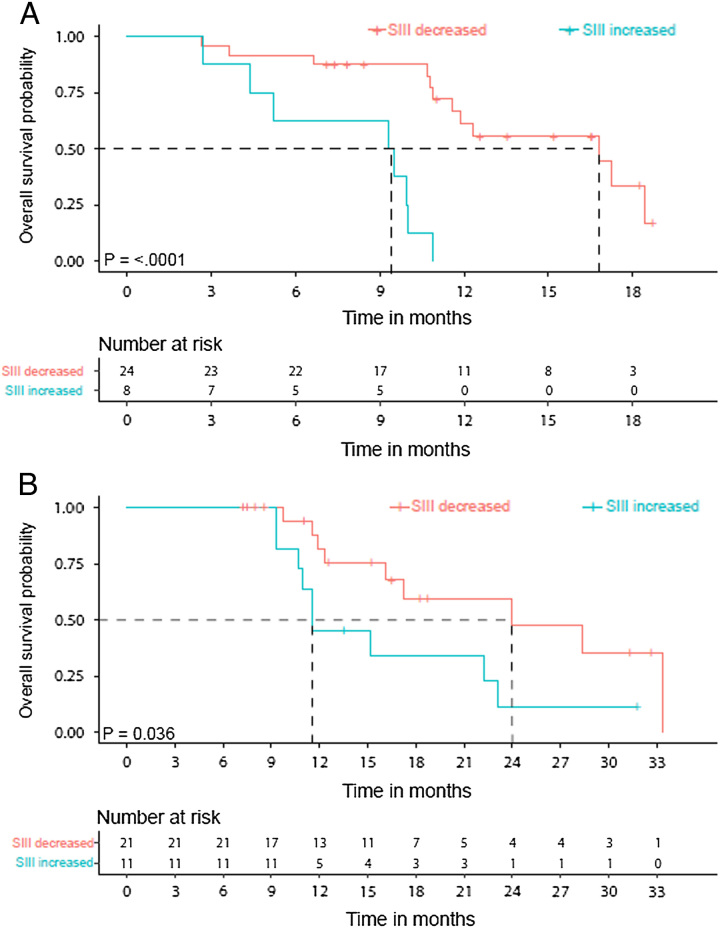
Increased SIII after treatment was associated with worse OS. A, Kaplan-Meier curves comparing OS of patients with increased SIII value after FOLFIRINOX compared with the baseline value (n = 8) versus OS of patients with decreased SIII value after FOLFIRINOX compared with baseline (n = 24). B, Kaplan-Meier curves comparing OS of patients with increased SIII value after FOLFIRINOX and SBRT compared with the baseline value (n = 11) versus OS of patients with decreased SIII value after FOLFIRINOX and SBRT compared with baseline (n = 21). The curves are statistically compared with a log-rank test.

Of the patients with an SIII value available at baseline and after FOLFIRINOX (n = 32), those with lowered SIII had a longer median OS than those with an increased SIII [16.8 (95% CI: 6.1–27.4) vs 9.3 (95% CI: 3.4–15.3) months; *P* < 0.0001; Fig. 3A]. Of the patients with an SIII value available at baseline and after SBRT (n = 32), patients with lowered SIII had a longer median OS than those with an increased SIII [24.0 (95% CI: 10.3–37.6) vs 11.6 (95% CI: 7.3–15.8) months; *P* < 0.036; Fig. 3B].

### Increase in Platelets After FOLFIRINOX Predicts Worse Overall Survival

A joint model analysis was performed to investigate the association between changes in the different components of the SIII and the instantaneous risk of dying. One ×10^9^/L increase in platelets increased the hazard ratio of dying by 1.007 (95% CI: 1.002, 1.011; *P* = 0.003). Changes in neutrophils (*P* = 0.454) and lymphocytes (*P* = 0.772) did not influence the hazard ratio of dying. In the patients with a platelet value available before and after FOLFIRINOX (n = 103), patients with increased platelet count had a worse median OS than those with lowered platelet count [4.5 (95% CI: 11.2–17.8) vs 9.5 (95% CI: 2.9–16.1) months; *P* < 0.009; Supplemental Digital Content Fig. 1A, http://links.lww.com/SLA/E514]. After FOLFIRINOX at time point 2, 20/117 (14.2%) patients had thrombocytopenia (ie, platelet count <00×10^9^/L). Patients with and without thrombocytopenia had equal survival outcomes (Supplemental Digital Content Fig. 1B, http://links.lww.com/SLA/E514).

### Carbohydrate Antigen 19-9 as a Prognostic Biomarker for Overall Survival During Treatment

In 132 patients, a CA 19-9 value was available at least 1 time point. In 105/132 (79.5%) patients, CA 19-9 was elevated at some point during their disease course. Thus, 27/132 (20.5%) of patients did not have elevated CA 19-9 levels. In 132 patients, the joint model analysis showed that one U/mL increase in log (CA 19-9) did not increase the instantaneous risk of dying. However, when only including 105 patients with elevated CA 19-9 in the analysis, one U/mL increase in log (CA 19-9) increased the hazard ratio of dying by 1.441 (95% CI: 1.166–1.781; *P* < 0.001).

### Resections

Twenty-four (17%) patients underwent an exploration and 20 (14.8%) patients underwent resection. The SIII values of the patients that underwent exploration but no tumor resection were 531, 885, and 345. The median SIII before the resection was 467 (IQR: 411–743) and the median CA 19-9 was 23 (IQR: 12–63) U/mL. In 12 of the resected patients, an SIII value at baseline and before the resection was available. In 11 of them, the SIII value was lower before the resection compared with the baseline (Supplemental Digital Content Table 3, http://links.lww.com/SLA/E515).

## DISCUSSION

In this retrospective study, we investigated the prognostic value of changes in the SIII during treatment with FOLFIRINOX chemotherapy alone, or FOLFIRINOX followed by SBRT, in patients with advanced pancreatic cancer. We found that the SIII significantly decreased after treatment with FOLFIRINOX chemotherapy. The SIII did not change after treatment with SBRT. Of note, there was a difference in the impact of SBRT on the SIII between patients. Also recently reported by others, there seems to be a clear individual difference in the development of radiation-induced lymphopenia after SBRT treatment.^22^ Most importantly, we found that an incline in SIII during treatment was associated with poor survival.

In addition to the SIII, other inflammatory biomarkers have been studied extensively in pancreatic cancer, and in cancer in general. A recent meta-analysis comprising 20 studies with 6512 patients, demonstrated the prognostic value of the modified Glasgow Prognostic Score, based on serum *C*-reactive protein and serum albumin levels, in the context of pancreatic cancer.^23^ Patients with high *C*-reactive protein levels and low albumin levels had a significantly worse prognosis, than those with normal levels.^23^ Furthermore, another meta-analysis demonstrated that a high neutrophil-to-lymphocyte ratio is associated with poor prognosis.^24^ Despite these promising results of different inflammatory biomarkers with regard to prognostication, they are not yet used in routine clinical decision-making. Currently, the tumor marker CA 19-9 is the most commonly used biomarker in PDAC during treatment.^10,11^ In our study, most of the included patients had elevated CA 19-9 levels at any time during their disease course. In these patients, CA 19-9 during treatment was demonstrated to be useful. However, 20.5% of patients included in our study did not have elevated CA 19-9 levels, which highlights the importance of finding additional biomarkers, especially in CA 19-9 nonproducers.

Unlike CA 19-9, the SIII was demonstrated to be a useful biomarker in all included patients. The exact mechanism underlying this observation is not clear, but several factors might play a role. First, emerging evidence suggests that neutrophils play an important role in carcinogenesis and cancer progression.^25,26^ Upon activation, neutrophils form neutrophil extracellular traps, which possibly increase the metastatic capability of tumor cells.^27^ Moreover, a recent study found that chemotherapy-induced tumor-infiltrating neutrophils can promote pancreatic cancer metastasis.^28^ Second, emerging evidence arises that tumor cells can actively use platelets to their advantage to promote cancer progression and metastasis.^29–31^ Another recent study demonstrated that pancreatic cancer cells acquire increased migration, invasion, and proliferation capabilities when brought in coculture with platelets.^32^ Furthermore, high intratumoral platelet infiltration is associated with worse recurrence-free survival and OS in patients with resected pancreatic cancer.^33^ Our data demonstrate that, next to an increase in the SIII, an increase in platelets during treatment is associated with worse survival. Third, lymphocytes, in contrast, possess the unique ability to induce an adaptive, antigen-specific antitumor immune response.^34^ Furthermore, a high lymphocyte, especially CD8+ T-cell infiltration in resected PDAC tumors, is associated with favorable survival outcomes.^35–37^ An increase in SIII through an increase of either neutrophils or platelets and/or a decrease in lymphocytes can logically result in a worse prognosis. An alternative explanation is, that it is not the change in the SIII that influences the disease course, but that an increase in SIII merely reflects cancer progression. Unfortunately, due to the observational and retrospective nature of our study, we were unable to draw definitive conclusions regarding precise underlying mechanisms.

The strength of the current study is the fact that data from all consecutive patients were analyzed. This minimized the risk of bias. Next to this, the joint model is an excellent model to investigate the relationship between a longitudinal blood biomarker and time-to-event data. In contrast, the current study has several limitations. First, an important limitation is its retrospective nature with its inherent missingness of data. In this respect, joint modeling is a reliable framework to address missing data as it has been shown to provide valid interference when data are missing completely at random (noninformative missingness) or when data are missing at random (missingness depends on information included in the model as survival for example). Complete data would, of course, have strengthened our findings. Second, at least 58.2% of patients used granulocyte-colony stimulating factor (G-CSF) as prophylaxis for chemotherapy-induced neutropenia and neutropenic fever. This percentage might have been even higher as in some cases G-CSF use may not have been documented adequately. G-CSF increases the proliferation and differentiation of neutrophils from progenitor cells.^38^ Also, it induces the maturation and function of mature neutrophils, resulting in a dose-dependent increase in neutrophils.^38^ Obviously, the administration of G-CSF influenced the values of SIII in our study. An increase in neutrophils through G-CSF could have resulted in an increase in the SIII. This is important as not all institutions use G-CSF as prophylaxis when treating PDAC patients with FOLFIRINOX, which possibly limits the representability of our data. Nevertheless, after treatment with FOLFIRINOX (with or without G-CSF), the SIII decreased significantly. Third, there is a notable difference between patients in intervals between blood drawings and treatment administrations. This was especially true for the laboratory values after SBRT, for which samples up to 100 days after SBRT were used. Fourth, the Dutch Pancreatic Cancer Group criteria for resectability differs slightly from the National Comprehensive Cancer Network criteria.^16,39^ Because of this, a proportion of the LAPC patients included in the study, would have been classified as borderline resectable pancreatic cancer according to the National Comprehensive Cancer Network criteria. Lastly, it would have been interesting to build a composite model with both the CA 19-9 and the SIII. However, due to the limited data (small number of measurements per patient and limited number of patients), we were not able to achieve model convergence. This model would have been able to also capture the correlation between CA 19-9 and SIII. Despite the limitations, this study clearly demonstrated the potential of the SIII as a longitudinal blood biomarker during treatment in advanced PDAC patients. A well-designed prospective trial, with repetitive, more frequent (ie, between every 2 cycles of FOLFIRINOX) blood draws during treatment is needed to confirm our findings. Currently, we are prospectively collecting SIII data in patients with resectable pancreatic cancer who are being treated with neoadjuvant FOLFIRINOX, with the aim to investigate whether an increase in SIII during neoadjuvant treatment is associated with a shorter recurrence-free and OS after resection in these patients.

## CONCLUSIONS

In addition to CA 19-9, the SIII is a reliable biomarker in patients with advanced pancreatic cancer during various types of treatment.

## Supplementary Material
