# Supplementary material for: Increasing Systemic Immune-inflammation Index During Treatment in Patients With Advanced Pancreatic Cancer is Associated With Poor Survival: A Retrospective, Multicenter, Cohort Study
Source: Ann Surg. 2023 Apr 3;278(6):1018–23. doi: 10.1097/SLA.0000000000005865 (PMC10631500; doi:10.1097/SLA.0000000000005865)
Supplement: Supplementary file 1 [file sla-278-01018-s001.docx]

| **Supplementary table 1** Laboratory data availability | | | | |
| --- | --- | --- | --- | --- |
| **Variable** | **Time point 1,**  **n (%)** | **Time point 2,**  **n (%)** | **Time point 3,**  **n (%)** | **At least one time point,**  **n (%)** |
| SIII | 89 (63.1) | 57 (40.4) | 56 (39.7) | 117 (83.0) |
| Neutrophils | 106 (75.2) | 112 (79.4) | 64 (45.4) | 132 (93.6) |
| Platelets | 122 (86.5) | 117 (83.0) | 71 (50.4) | 136 (96.5) |
| Lymphocytes | 90 (63.8) | 57 (40.4) | 56 (39.7) | 117 (83.0) |
| Leukocytes | 113 (80.1) | 110 (78.0) | 69 (48.9) | 128 (90.8) |
| CA 19-9 | 115 (81.6) | 70 (49.6) | 61 (43.3) | 132 (93.6) |
| CEA | 93 (66.0) | 86 (61.0) | 58 (41.1) | 125 (88.7) |
| Bilirubin | 128 (90.8) | 109 (77.3) | 66 (46.8) | 137 (97.2) |
| CRP | 86 (61.0) | 26 (18.4) | 27 (19.1) | 101 (71.6) |
| *Data are shown as counts with percentages. SIII = Systemic Immune-Inflammation index, CA 19-9 = Carboydrate antigen 19-9, CEA = Carcinoembryonic antigen, CRP = C-reactive protein.* | | | | |
