# Supplementary material for: Increasing Systemic Immune-inflammation Index During Treatment in Patients With Advanced Pancreatic Cancer is Associated With Poor Survival: A Retrospective, Multicenter, Cohort Study
Source: Ann Surg. 2023 Apr 3;278(6):1018–23. doi: 10.1097/SLA.0000000000005865 (PMC10631500; doi:10.1097/SLA.0000000000005865)
Supplement: Supplementary file 2 [file sla-278-01018-s002.docx]

| **Supplementary table 2** Median SIII, neutrophil, platelet and lymphocyte counts on the different time points | | | | | | |  |  |
| --- | --- | --- | --- | --- | --- | --- | --- | --- |
|  | | **Time point 1** | | **Time point 2** | | **Time point 3** | | |
| **Variable** | **n (%)** | **Median (IQR)** | **n (%)** | **Median (IQR)** | **n (%)** | **Median (IQR)** |  |  |
| SIII | 89 (63.1) | 908 (631 – 1276) | 57 (40.4) | 488 (352 – 692) | 56 (61.5) | 536 (398 – 832) |  |  |
| Neutrophil count (× 10^9/^L) | 106 (75.2) | 5.23 (4.19 – 6.73) | 112 (79.4) | 5.91 (2.97 – 10.52) | 64 (70.3) | 3.20 (2.53 – 4.28) |  |  |
| Platelet count (× 10^9/^L) | 122 (86.5) | 277 (212 – 330) | 117 (83.0) | 174 (123 – 174) | 71 (78.0) | 190 (131 – 233) |  |  |
| Lymphocyte count (× 10^9/^L) | 90 (63.8) | 1.53 (1.26 – 2.10) | 57 (40.4) | 1.49 (1.55 – 1.99) | 56 (61.5) | 0.96 (0.68 – 1.44) |  |  |
| *n = number of patients in which the variable is available at each time point, with the associated percentage (%). Data are shown as medians with interquartile range. SIII = Systemic Immune –Inflammation Index, IQR = interquartile range.* | | | | | | |  |  |
