# Supplementary figures and images for: Increasing Systemic Immune-inflammation Index During Treatment in Patients With Advanced Pancreatic Cancer is Associated With Poor Survival: A Retrospective, Multicenter, Cohort Study
Source: Ann Surg. 2023 Apr 3;278(6):1018–23. doi: 10.1097/SLA.0000000000005865 (PMC10631500; doi:10.1097/SLA.0000000000005865)

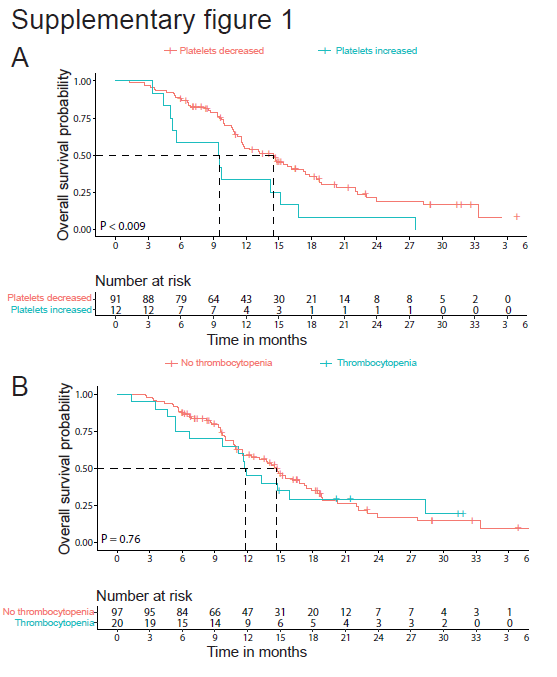

Supplement: Supplementary file 3 [file sla-278-01018-s003.tif]
