# Supplementary material for: Increasing Systemic Immune-inflammation Index During Treatment in Patients With Advanced Pancreatic Cancer is Associated With Poor Survival: A Retrospective, Multicenter, Cohort Study
Source: Ann Surg. 2023 Apr 3;278(6):1018–23. doi: 10.1097/SLA.0000000000005865 (PMC10631500; doi:10.1097/SLA.0000000000005865)
Supplement: Supplementary file 4 [file sla-278-01018-s004.docx]

| **Supplementary table 3** SIII values of the resected patients | | | |
| --- | --- | --- | --- |
| **Subject** | **Time point 1** | **Time point 2** | **Time point 3** |
| SIII001 | - | - | - |
| SIII017 | - | - | - |
| SIII029 | - | - | 644 |
| SIII030 | - | - | 1032 |
| SIII037 | 810 | 589 | **467** |
| SIII040 | 2186 | 529 | **540** |
| SIII041 | - | 457 | 458 |
| SIII043 | 965 | 439 | **781** |
| SIII045 | - | 415 | 416 |
| SIII051 | 428 | 241 | **197** |
| SIII065 | 850 | 391 | **704** |
| SIII069 | 1125 | - | **513** |
| SIII071 | 814 | - | 1174 |
| SIII087 | 1187 | 287 | **461** |
| SIII088 | - | 281 | 174 |
| SIII102 | 675 | - | **405** |
| SIII113 | 615 | - | **318** |
| SIII118 | 858 | - | **418** |
| SIII136 | 821 | - | **-** |
| SIII137 | 912 | **842** |  |
| *SIII = Systemic Immune-Inflammation Index. Blank cells are missing values. Bold values represent SIII values that are lower before the resection than at baseline. Shaded cells illustrate subjects with SIII values available at baseline and before the resection.* | | | |
